# Heat Stress Detection in Dairy Cattle Using Intraruminal Bolus Sensors

**DOI:** 10.3390/ani16162516

**Published:** 2026-08-12

**Authors:** Levente Kovács, Áron Gergely Simon, Martin Czirok, Péter Póti, Viktor Jurkovich

**Affiliations:** 1 Institute of Animal Sciences, Gödöllő Campus, Hungarian University of Agriculture and Life Sciences, Páter Károly utca 1, H-2100 Gödöllő, Hungary; czirok.martin@uni-mate.hu (M.C.); poti.peter@uni-mate.hu (P.P.); 2 Hunland Dairy Ltd., Alsóráda puszta 13, H-2347 Bugyi, Hungary; simiaron@gmail.com; 3 Centre for Animal Welfare, University of Veterinary Medicine, István u. 2, H-1078 Budapest, Hungary; jurkovich.viktor@univet.hu

**Keywords:** intraruminal bolus sensor, heat stress, dairy cattle, reticuloruminal temperature, machine learning, precision livestock farming, LoRaWAN

## Abstract

Heat stress represents one of the most economically and biologically consequential environmental challenges facing modern dairy production, with projected intensification under ongoing climate change. Intraruminal bolus sensors have emerged as a uniquely capable platform for heat stress surveillance in lactating dairy cows: once administered, they provide continuous, lifetime access to reticuloruminal temperature—a reliable indicator of core body thermal status—alongside behavioral parameters, in a configuration entirely shielded from external environmental interference. This review examines the scientific basis and practical capabilities of bolus-based heat stress monitoring and its potential to optimize dairy production systems. We describe the physiological rationale for reticuloruminal temperature as a heat stress indicator, the principal confounding factors including drinking-water effects and diurnal variation, and the evidence base for interpreting temperature signals in the context of breed, parity, milk yield, and the temperature–humidity index. We review controlled studies characterizing the determinants of reticuloruminal temperature across breeds and seasons, and a four-level machine-learning processing architecture for bolus data integration. Individual baseline calibration—rather than fixed population thresholds—is identified as a prerequisite for reliable detection, enabling targeted cooling interventions, improved reproductive management, and enhanced disease surveillance. We further review bolus developments extending sensing to heart-rate monitoring and low-power wide area network communication, and discuss machine-learning methodologies suited to bolus-derived time-series data. Methodological challenges—including drinking-water effects, sensor precision, communication reliability, and the absence of standardized crossbreed validation protocols—are critically appraised, and future research priorities are identified.

## Simple Summary

Heat stress poses a major economic and welfare challenge in modern dairy farming, impairing milk production, reproductive efficiency, and immune function in lactating cows. Current farm management relies primarily on the temperature–humidity index, an environmental metric that does not reflect the physiological state of individual animals. Intraruminal bolus sensors, permanently retained in the reticulum after a single oral administration, provide continuous access to reticuloruminal temperature alongside behavioral parameters including activity, rumination, and drinking frequency; a latest-generation prototype additionally incorporates pulse-rate monitoring. When combined with individual baseline calibration and machine-learning analytics, these sensors enable earlier and more accurate heat stress detection than environment-based monitoring alone. They can distinguish heat stress from fever and estrus-related temperature elevations. This review synthesizes the physiological rationale for reticuloruminal temperature monitoring, the evidence-based characterization of its determinants and confounders, and the hardware and analytical advances that are bringing bolus-based heat stress detection closer to routine farm application.

## 1. Introduction

The global dairy industry faces mounting pressure from thermally challenging climates, with economic losses attributable to heat stress in the United States alone estimated between USD 897 million and USD 1.5 billion annually [1,2]. Climate-driven intensification of heat stress is expected to reduce global milk production substantially through 2100, with the most severe impacts concentrated in tropical and subtropical regions [3]. Against this background, accurate, timely, and individual-level detection of heat stress has become a central objective for precision livestock farming (PLF) research.

The temperature–humidity index (THI), computed from ambient dry-bulb temperature and relative humidity, is the standard operational descriptor of the bovine thermal environment. Threshold values of THI > 68, 72, and 80 are commonly associated with mild, moderate, and severe heat stress in Holstein cows, respectively, though high-producing cows may begin showing physiological responses at even lower values [4,5]. However, the THI captures environmental conditions at a single spatial point; it says nothing about the physiological state of the individual animal, which is shaped by breed, body mass, milk yield, health status, acclimatization, and access to shade and water [6,7]. Individual biosensors capable of registering the physiological expression of thermal stress—rather than merely the environmental conditions that may induce it—are therefore essential for accurate, animal-centered heat stress monitoring.

**Figure 1:**
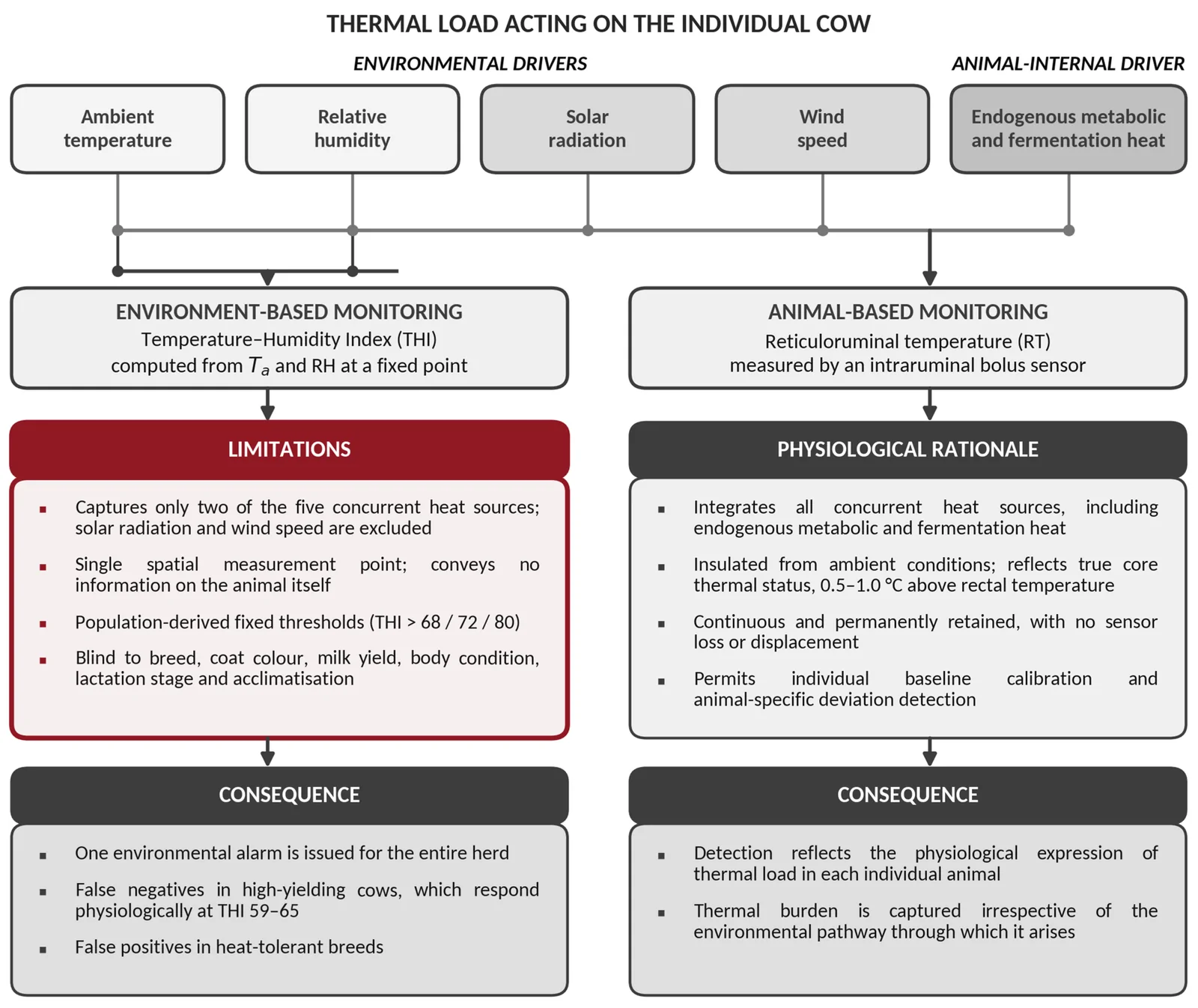
The limitations of environment-based thermal assessment and the physiological rationale for individual, animal-based heat stress monitoring. The authors’ own conceptual synthesis based on [4,5,6,7,8,9,10,11,12,13,14,15,16,17].

The THI was originally derived from population-level studies and does not account for substantial inter-individual variation in the physiological expression of thermal stress, which arises from breed, coat color, acclimatization history, body condition, and lactation stage [8]. High-producing Holstein cows at peak lactation may exhibit measurable physiological heat stress responses at THI values as low as 59–65 [4,9,10], well below the conventionally applied threshold of 68 [9]. At the same time, heat-tolerant breeds maintain core temperature stability at values that would severely compromise Holstein cattle [11]. However, THI does not incorporate solar radiation or wind speed, both of which substantially modulate the effective thermal load on animals, particularly in pasture-based systems. More comprehensive indices—including THI adjusted for solar radiation and wind speed, and the Comprehensive Climate Index, which integrates temperature, humidity, wind speed, and solar radiation—have been proposed to better characterize thermal stress in grazing conditions [12,13]. Since reticuloruminal temperature (RT) reflects the integrated physiological response to all concurrent heat sources—ambient temperature, humidity, solar radiation, wind speed, and endogenous metabolic heat production—bolus-based monitoring inherently captures the full thermal burden experienced by the individual animal, irrespective of the environmental pathway through which that burden is imposed [14]. This capacity is relevant in pasture-based and naturally ventilated systems, where solar radiation load may represent the dominant driver of heat stress yet remains invisible to standard THI-based monitoring [14]. These limitations, summarized together with the corresponding physiological rationale in Figure 1, constitute a compelling scientific and practical rationale for animal-centered, physiological monitoring as a complement or replacement for environment-only THI surveillance. The central thesis of this review is that intraruminal bolus sensors, combined with individual baseline calibration and machine learning, provide a technically mature platform for achieving this objective.

Among available PLF sensors, the intraruminal bolus occupies a distinctive position. Administered orally and permanently retained in the reticulum, it provides continuous, stable access to the reticulorumen compartment without the sensor loss, displacement, or effects that limit wearable devices [15,16]. Reticuloruminal temperature exceeds rectal temperature by approximately 0.5–1.0 °C due to microbial fermentation heat, is fully insulated from ambient conditions, and therefore reflects true core thermal status rather than surface or environmental temperature [15,17]. Modern multi-sensor boluses should be able to further extend this capability to accelerometry, rumination detection, drinking behavior identification, and, in special cases, heart-rate measurement [16].

This review provides a focused synthesis of the scientific foundations, technological state of the art, and analytical approaches for bolus-based heat stress detection in dairy cattle. We cover the physiological rationale for RT monitoring, the evidence-based characterization of its determinants and confounders, the machine-learning processing architecture applicable to bolus data, and recent multiparametric hardware developments. Methodological challenges and future research priorities are also addressed. While intraruminal boluses have been available for some years as physiological monitoring devices, their application to heat stress detection has only recently been transformed by three concurrent developments: the integration of machine-learning algorithms capable of handling the high-dimensional, time-series data streams from next-generation multiparametric boluses; the emergence of PLF as an operational framework that contextualizes individual sensor outputs within herd-level management decisions; and growing recognition of heat stress as a key constraint on sustainable intensification in both housed and pasture-based dairy systems. This review is therefore timely as it synthesizes these developments into a coherent framework for applying bolus-based, artificial intelligence (AI)-enabled heat stress monitoring.

## 2. Methods

The literature search was performed independently by two authors in the Web of Science (Core Collection), Scopus, and PubMed databases, covering publications from January 2000 to December 2025. The search was supplemented by manual screening of the reference lists of all included articles and of relevant review papers identified during the search.

The following search string was applied across title, abstract, and keyword fields: (“intraruminal bolus” OR “rumen bolus” OR “reticuloruminal temperature” OR “rumen sensor”) AND (“heat stress” OR “thermal stress” OR “temperature-humidity index” OR “THI”) AND (“dairy cow” OR “dairy cattle” OR “lactating cow” OR “Holstein”). Additional searches were performed with the terms “rumen pH bolus”, “machine learning heat stress cattle”, “random forest bovine temperature”, “LSTM dairy cattle”, and “precision livestock farming heat stress” to capture the algorithm-focused and technology-focused literature.

Studies were included if they (i) used at least one intraruminal bolus sensor as the primary measurement device for reticuloruminal temperature and/or pH; (ii) reported original data collected from lactating dairy cattle; (iii) addressed heat stress detection, thermal load quantification, or a physiological response to elevated ambient temperature; and (iv) were published in a peer-reviewed journal or presented a validated machine-learning or algorithmic framework with sufficient methodological detail for assessment. A single, explicitly identified exception to criterion (iv) was made for one unpublished manuscript by the present authors [18]. It is included for contextual purposes only, it does not contribute to any quantitative statement in this review, and it is identified as unpublished wherever it is cited. Reviews, conference abstracts, and gray literature were excluded from the primary evidence synthesis but were considered for contextual and conceptual discussion where relevant.

Studies were excluded if they (i) used rectal, vaginal, or tympanic temperature sensors exclusively, without bolus data; (ii) focused exclusively on thermoneutral conditions without any thermal challenge; (iii) reported only simulation or modeling results without empirical bolus data; or (iv) were not available in English.

**Figure 2:**
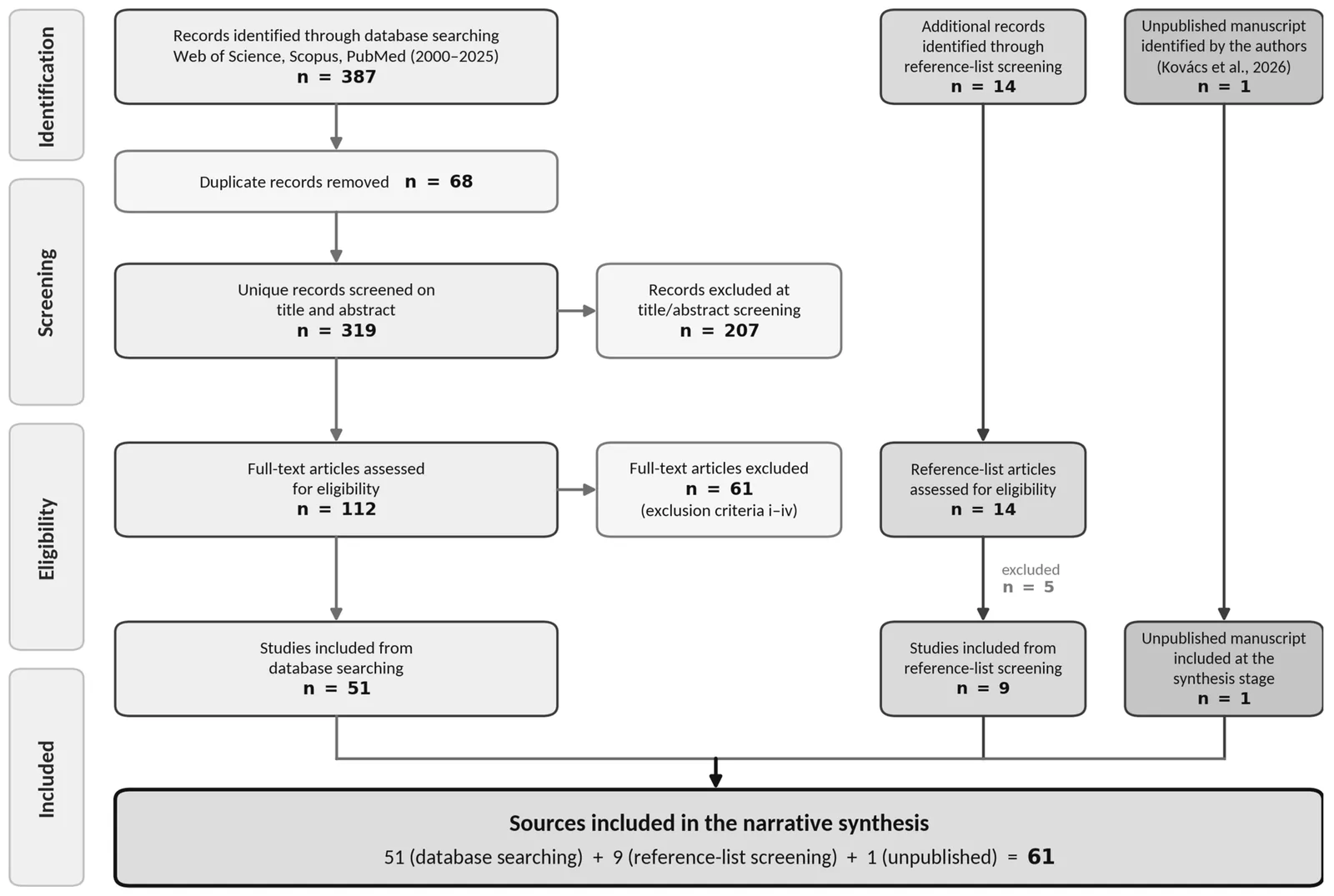
A flow diagram of the literature search and study selection process [18].

The initial search retrieved 387 records. After deduplication (68 duplicate records removed), 319 unique records remained, of which 207 were excluded at the title and abstract screening stage. Full-text assessment was performed for 112 articles, of which 61 were excluded and 51 met all inclusion criteria and were included in the final evidence synthesis. An additional 14 articles identified through reference list screening were assessed, of which five were excluded and nine were included. One further source, an unpublished manuscript by the present authors [18], was added directly at the synthesis stage. The final corpus therefore comprises 61 sources: 60 peer-reviewed publications and one unpublished manuscript. Data extraction covered the study design, sample size, breed and production level, bolus type and measurement interval, heat stress definition (THI threshold used), statistical or machine-learning method, key RT and/or pH findings, and reported performance metrics where applicable. A narrative synthesis was adopted given the heterogeneity of study designs, outcome measures, and analytical approaches across the included literature. Table 1 and Table 2 summarize the evidence from the included studies on published RT/pH heat stress findings and on bolus platforms, respectively. The complete study selection process is illustrated in Figure 2. The present work is framed as a narrative review rather than a systematic review; no protocol was registered, no formal risk-of-bias appraisal was undertaken, and no quantitative pooling was attempted, because the heterogeneity of study designs, bolus platforms, heat stress definitions and outcome measures across the included literature precluded meaningful meta-analysis. PRISMA is therefore applied here as a reporting framework for the literature search, not as a claim of systematic-review methodology.

## 3. Physiological Basis of Core Body Temperature Monitoring

### 3.1. Thermoregulatory Failure and Its Consequences

Dairy cows maintain core body temperature within a narrow range—approximately 38.0–39.3 °C—through a balance between endogenous metabolic heat production and dissipation to the environment [27]. High-producing lactating cows generate a disproportionate endogenous heat burden due to the intense metabolic activity associated with milk synthesis: a cow producing 40 kg of milk daily generates roughly 30% more metabolic heat than a dry cow of comparable body mass [27]. When the environmental heat load approaches or exceeds the dissipation capacity, a cascade of thermoregulatory responses ensues: increased respiratory rate, peripheral vasodilation, reduced dry matter intake, decreased physical activity, and extended standing time to maximize body surface area exposed to convective cooling [28,29].

The economic and biological consequences are far-reaching. Milk yield, composition, and quality parameters (fat percentage, protein content, and somatic cell count) decline, with roughly 35–50% of the production loss attributable to direct, feed-intake-independent effects on mammary physiology, including altered insulin signaling and impaired glucose partitioning [28]. Reproductive efficiency is severely compromised: elevated core temperature disrupts oocyte maturation, early embryonic development, and the luteolytic cascade, lowering first-service conception rates by 15–20 percentage points in summer versus winter [30,31]. Systemic effects—oxidative stress, immune suppression, and leaky-gut-associated endotoxemia—further amplify the clinical and economic impact [32,33].

An underappreciated component of this burden in high-producing cows is ruminal fermentation, which contributes to total endogenous heat production: heat generated as a by-product of microbial volatile fatty acid synthesis and protein turnover accounts for roughly 15–25% of total body heat on high-forage diets [27]. During heat stress, voluntary dry matter intake typically falls by 10–25% [34], lowering the fermentation heat load but also the energy available for milk synthesis—hence the characteristic dissociation between modestly reduced intake and disproportionately greater milk yield loss that Baumgard and Rhoads [28] attributed to a direct, intake-independent effect on mammary metabolism. The accompanying drop in rumination duration is detectable by tri-axial accelerometry in modern multi-sensor boluses, providing a behavioral correlate of this metabolic adaptation to thermal load that complements the direct RT signal.

**Figure 3:**
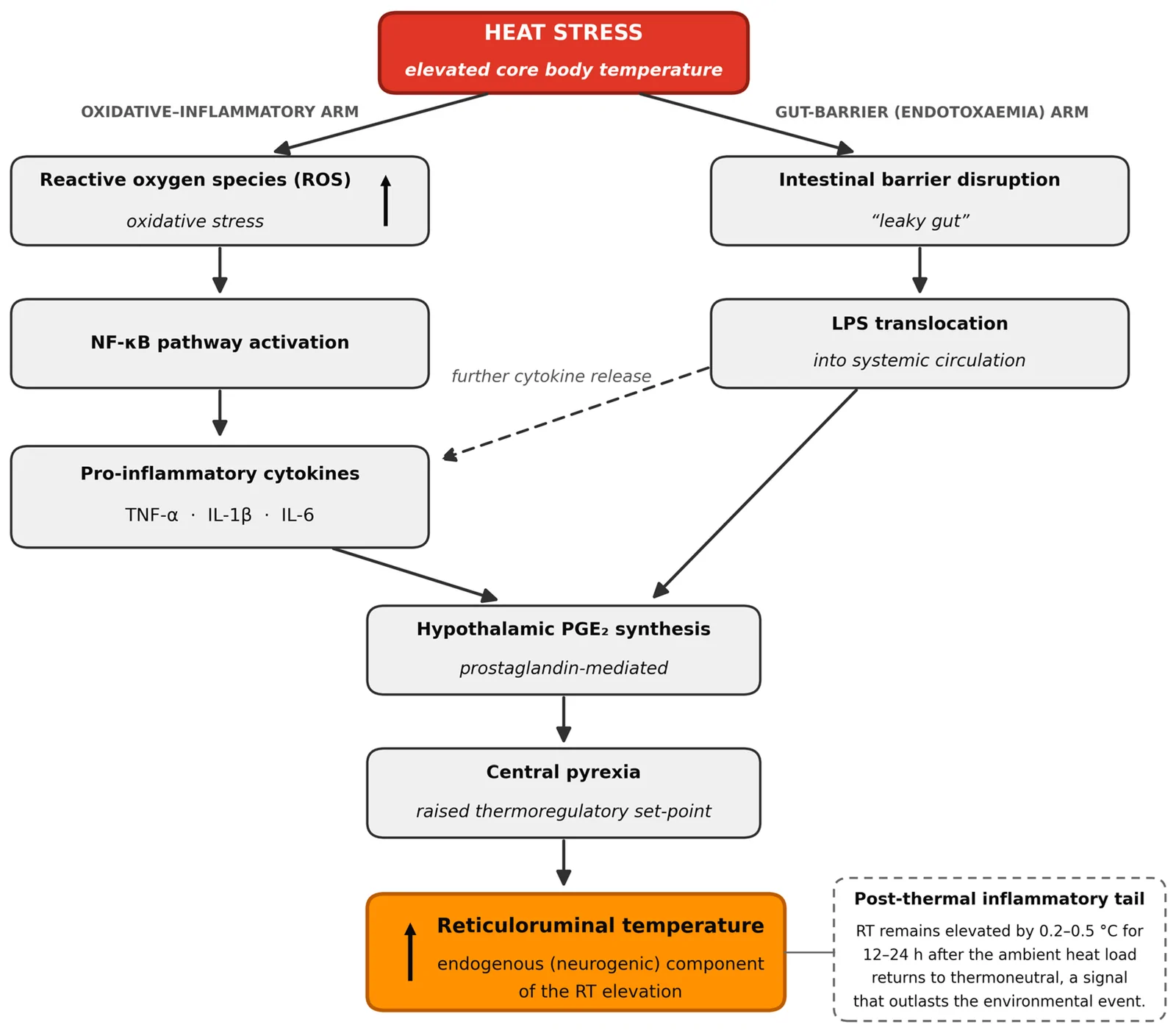
Endogenous inflammatory–pyrexic pathway contributing to reticuloruminal temperature elevation during heat stress. Authors’ own conceptual synthesis based on [32,33,35,36,37].

Beyond the direct thermoregulatory cascade, heat stress initiates a systemic inflammatory response with direct implications for the interpretation of reticuloruminal temperature signals. Elevated core temperature promotes the generation of reactive oxygen species, triggering oxidative stress that compromises cellular antioxidant defenses and activates the nuclear factor-κB pathway, leading to the release of pro-inflammatory cytokines (TNF-α, IL-1β, IL-6) [35]. This inflammatory activation independently elevates core body temperature through prostaglandin-mediated central pyrexia, contributing to the RT elevation during heat stress and making it neurogenic rather than purely thermogenic in origin [36]. Concurrently, heat stress increases intestinal permeability—the so-called “leaky gut” phenomenon—thereby facilitating the translocation of lipopolysaccharide (LPS) from Gram-negative bacteria into the systemic circulation [32,33]. Circulating LPS stimulates further cytokine release and hypothalamic prostaglandin synthesis, amplifying the febrile component of the RT elevation [37]. This means that during sustained heat stress, the RT signal reflects not only the thermoregulatory equilibrium between environmental heat load and dissipation capacity, but also an endogenous inflammatory–pyrexic contribution that may persist even after the ambient THI returns to thermoneutral levels. Algorithms designed to detect the cessation of heat stress events using RT alone must therefore account for this post-thermal inflammatory tail, which can sustain RT elevations of 0.2–0.5 °C for 12–24 h beyond the end of the environmental heat event [36,37]. These two convergent arms—an oxidative–inflammatory arm and a gut-barrier (endotoxaemic) arm—and their integration at the level of hypothalamic prostaglandin E2 synthesis are summarized in Figure 3.

### 3.2. Reticuloruminal Temperature as a Core Body Thermal Indicator

The physiological validity of RT as an indicator of core body thermal status rests on three established observations. First, RT correlates significantly with rectal temperature: Bewley et al. [15] documented a consistent relationship between the two measures, with RT exceeding rectal temperature by 0.5–1.0 °C due to the exothermic activity of rumen microorganisms. The correlation between RT and THI varies across studies, ranging from r = 0.22 to r = 0.645 [15,20,25]. Second, RT responds to heat load in a manner consistent with core temperature elevation, with a significant positive association between rising THI and increasing RT across all breeds studied [8]. Third, the bolus is shielded from the external thermal environment, making RT measurements unaffected by the ambient temperature, solar radiation, or coat conditions that confound surface-mounted sensor readings [16,17]. The combination of internal sensor placement and a correlation with established reference measures provides RT with a physiological rationale for serving as the primary heat stress signal in bolus-based monitoring systems. The low-to-medium correlation between RT and THI would be an influencing factor. In addition, there are some confounders and limitations when measuring RT that will be discussed in the following sections.

### 3.3. Measurement Confounders: Drinking Water, Feeding, and Environmental Determinants

Despite its advantages, RT is subject to several sources of variation that must be controlled for reliable interpretation of heat stress. The most impactful is drinking-water ingestion. Bewley et al. [15] showed that drinking cold water can depress RT by up to 8.5 °C, with recovery to baseline requiring up to 2 h. During heat stress, water intake frequency and volume increase substantially, raising the risk of systematic underestimation of core temperature precisely when heat stress is most severe. Vázquez-Diosdado et al. [38] developed and validated a threshold-based algorithm capable of detecting drinking episodes from the characteristic RT signal profile, enabling post hoc correction; Lees et al. [39] demonstrated that the drinking-water-corrected adjusted reticuloruminal temperature (ART) substantially outperformed raw RT as a heat stress event predictor in a field study of 443 grazing cows.

Feeding-induced fermentation generates a transient RT elevation of 0.3–0.7 °C in the 1–2 h following feeding, depending on forage-to-concentrate ratio and meal size [15]. A circadian rhythm of approximately 0.3–0.5 °C amplitude has been documented, with RT nadir at approximately 10:00 and acrophase at approximately 23:30 in multiple independent studies [7,19]. These systematic variations impose an absolute requirement for time-of-day normalization in any analytical pipeline that aims to detect heat stress events against a physiological background. Commercial bolus sensor accuracy is typically ±0.1–0.3 °C, which is sufficient for fever detection but marginal for reliably capturing the smaller RT elevations—0.3–0.8 °C—associated with estrus or mild early heat stress [17].

The interaction between heat stress and feeding-induced fermentation creates a particularly challenging interpretive problem that has received insufficient attention in the bolus literature. During heat stress, cows shift their feeding behavior towards night-time eating to exploit cooler ambient temperatures [34]; this temporal redistribution of dry matter intake generates fermentation-associated RT peaks that are phase-shifted relative to the thermoneutral feeding schedule, potentially compounding or masking the heat-stress-induced RT elevation during the same nocturnal period. Algorithms trained on thermoneutral feeding schedules will therefore misinterpret RT peaks in heat-stressed cows unless they explicitly account for the interaction between heat load, feeding time, and fermentation dynamics—a methodological gap that current published correction algorithms [38] do not fully address. The limitations of current correction approaches are threefold. First, published algorithms are threshold-based and single-confounder-oriented: they identify drinking episodes from a stereotyped drop-and-recovery profile [38] and implicitly assume that other sources of RT variation act additively and independently, thus overlapping drinking, feeding, and thermal events within the same window cannot be separated. Second, their thresholds and recovery windows are derived from specific herds, housing systems, and water temperatures and validated under thermoneutral or moderately warm conditions, leaving transferability to high-THI periods—when drinking frequency, water temperature, and feeding times shift simultaneously—largely untested. Third, correction is applied post hoc at the population level, without individual calibration or a concurrent core temperature reference, so over-correction may remove part of the genuine heat stress signal and the accuracy of corrected values cannot be quantified in the field. Additionally, Polsky and von Keyserlingk [8] highlight that heat stress substantially alters lying behavior—heat-stressed cows spend more time standing to maximize heat dissipation from the udder and legs—and this shift is independently detectable by the accelerometry channel of multi-sensor boluses, providing a non-thermal corroborating signal for heat stress that is not subject to the drinking-water or fermentation confounders that affect RT. Furthermore, cooling mitigation strategies—including sprinkler systems, fan ventilation, and provision of shade—directly reduce RT by lowering effective ambient heat load; these interventions must be considered as potential confounders when interpreting RT data, as they can suppress RT below the expected heat stress elevation and create temporal effects in continuous monitoring records. Wind speed similarly reduces the effective thermal load on the animal surface. It should ideally be incorporated into environmental covariates alongside THI when interpreting bolus-derived RT in pasture-based systems. While behavioral signals (lying time, rumination duration, drinking frequency) provide valuable corroborating evidence for heat stress, RT offers a critical physiological anchor that behavioral proxies cannot replace: it directly quantifies the degree of thermoregulatory failure at the individual level, enabling personalized intervention thresholds and detection of subclinical thermal stress before overt behavioral changes manifest. Behavioral responses also exhibit substantial individual and diurnal variation independent of thermal load, reducing their standalone specificity as heat stress classifiers [40]. The key empirical findings from studies characterizing these confounders are summarized in Table 1.

### 3.4. Circadian Dynamics of Reticuloruminal Temperature and pH During Heat Stress

**Figure 4:**
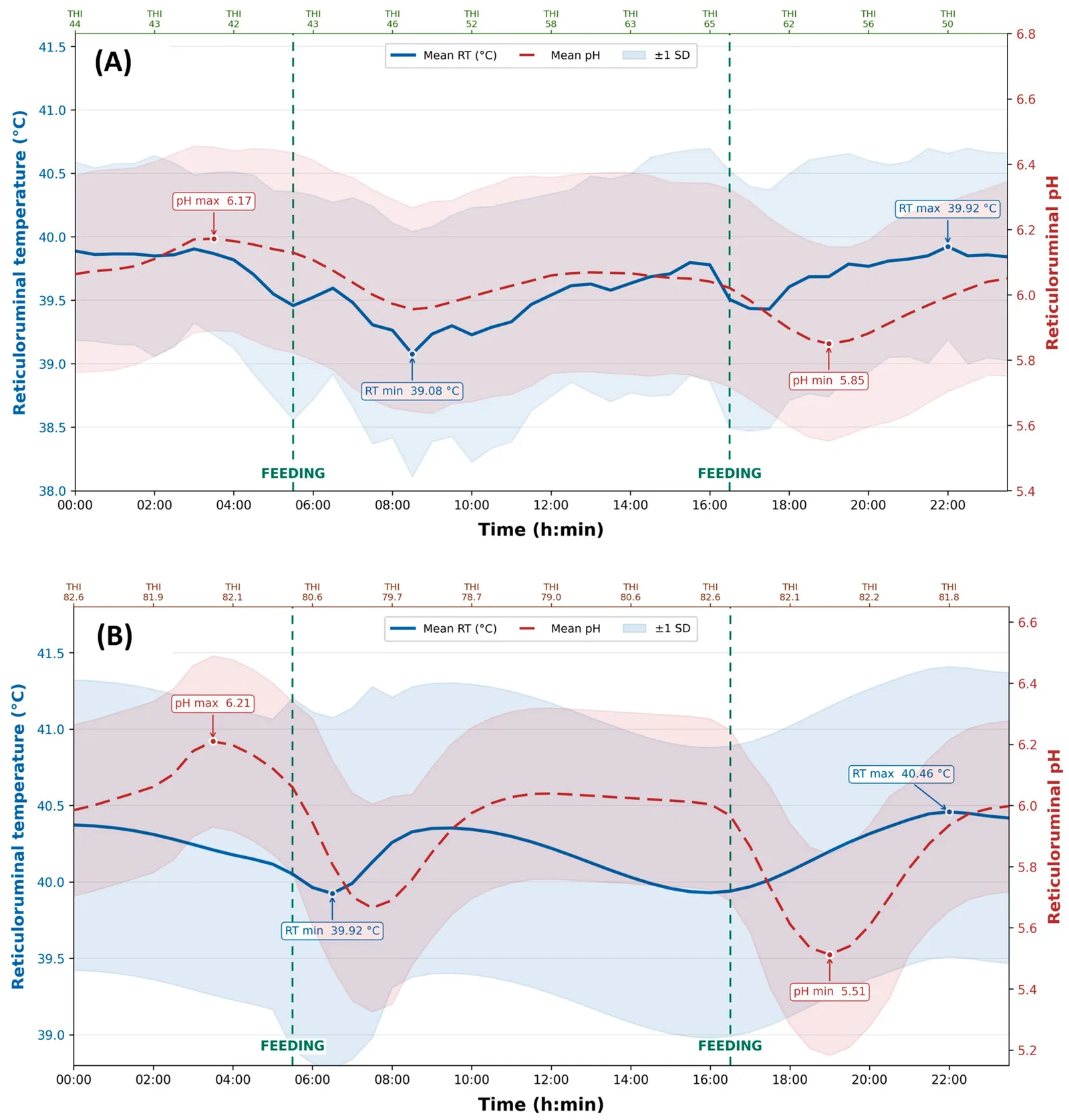
The mean (±SD) circadian profiles of reticuloruminal temperature and reticuloruminal pH recorded by intraruminal wireless bolus sensors in 18 Holstein–Friesian dairy cows under (**A**) thermoneutral conditions (THI range: 42.0–65.2) and (**B**) heat stress episode (THI range: 75.9–83.6). The estimated THI values are shown along the upper axis [18]. Solid lines show the hourly mean of reticuloruminal temperature (blue, left axis) and reticuloruminal pH (red, right axis). The shaded bands denote ±1 standard deviation around the corresponding mean and therefore represent between-animal variation at each time point. Vertical dashed lines mark the two feeding events, and the upper axis gives the concurrent temperature–humidity index (THI).

Figure 4 illustrates the 24 h circadian profiles of reticuloruminal temperature (RT) and pH recorded under thermoneutral and heat stress conditions using wireless intraruminal bolus sensors in 18 Holstein–Friesian dairy cows [18]. Both parameters display pronounced circadian rhythmicity that closely mirrors published findings from independent cohorts [7,19,21]. Under thermoneutral conditions (Figure 4A; estimated THI range: 42–65), RT follows a smooth daily cycle with a nadir at approximately 08:30 h and an acrophase at approximately 22:00 h, consistent with the pattern documented by Stone et al. [7] and Liang et al. [19] across Holstein, Jersey, and crossbred cattle. The RT nadir coincides with the post-feeding drinking event: ingestion of large volumes of cold water transiently depresses reticuloruminal temperature for up to 120 min [15]. Cantor et al. [24] similarly showed that the quantity and temperature of ingested water significantly affect both the magnitude and duration of this RT depression. They proposed that algorithmic correction for drinking events is essential for reliable heat stress detection [24]. Reticuloruminal pH peaks at approximately 03:30 h during the overnight recovery phase, when fermentative activity is lowest and salivary bicarbonate buffering has partially restored ruminal pH, as described by Humer et al. [41] and Neubauer et al. [42]. After the morning feeding, pH declines rapidly, partially recovers, and reaches its daily minimum at approximately 19:00 h after afternoon feeding (16:30 h), reflecting a biphasic post-prandial fermentation pattern. This diurnal pH profile, including its characteristic post-feeding nadirs, is consistent with data from wireless bolus monitoring studies in commercial dairy herds reported by Antanaitis et al. [22,23]. Across multiple independent studies, the nadir of RT under thermoneutral conditions is consistently reported in the range of 38.4–38.9 °C, occurring between 08:00 and 11:00 h, while the acrophase reaches 39.1–39.7 °C between 21:00 and 24:00 h, corresponding to the period of maximal fermentation activity following the main evening feeding event. Under sustained heat stress, both the nadir and acrophase shift upward by 0.3–0.8 °C, and the amplitude of the circadian oscillation may increase or decrease depending on the severity of thermal load and the animals’ access to cooling resources.

Under sustained heat stress (Figure 4B; THI range: 75.9–83.6, all values above the threshold of 72 throughout the entire 24 h period), both parameters are markedly altered relative to thermoneutral conditions [18]. RT is elevated by approximately 0.4–0.5 °C across the full diel cycle, in close agreement with the 0.3–0.8 °C elevation reported by Stone et al. [7] for Holsteins exposed to THI values above 65, and with the findings of Ammer et al. [20], who documented a significant positive correlation between daily median RT and THI above 65 in a multi-farm German cohort. The RT maximum shifts to approximately 22:00 h, tracking the genuine THI maximum recorded inside the closed barn: the THI nadir paradoxically occurs in the afternoon (∼16:30 h, THI ∼75.9) and the maximum at night (∼22:30 h, THI ∼83.6), a pattern characteristic of heat-loaded livestock buildings where daytime ventilation partially mitigates the thermal burden but nocturnal radiant and convective heat from the building fabric and the animals themselves accumulates and cannot dissipate. The drinking effect at morning feeding remains present but is proportionally smaller relative to the elevated baseline RT. Reticuloruminal pH is overall lower under heat stress (∼5.51–6.21 vs. ∼5.85–6.18 under thermoneutral conditions), and the post-feeding pH drops are markedly steeper and deeper; the afternoon nadir of ∼5.51 falls substantially below the conventional wireless-bolus SARA threshold of pH 6.0 [42] or pH 5.8 applied to ventral rumen sac samples [41]. Antanaitis et al. [22] reported analogous findings using smaXtec boluses in a Lithuanian commercial herd, documenting that THI ≥ 72 was associated with significantly lower reticuloruminal pH, reduced rumination index, and increased locomotor activity relative to thermoneutral periods, confirming the co-occurrence of acidosis risk and behavioral heat stress responses. The reduced post-feeding pH recovery under heat stress is mechanistically consistent with the well-documented reduction in salivary flow and bicarbonate secretion during thermal challenge [27,34], which diminishes ruminal buffering capacity precisely when the fermentation acid load following concentrate meals is highest.

## 4. Individual Modulation of Reticuloruminal Temperature

### 4.1. Breed, Milk Yield, Parity, Calving, and Disease

The transition period surrounding parturition introduces additional modulation of RT that must be accounted for in heat stress interpretation algorithms. Periparturient physiological immunosuppression, negative energy balance, and the inflammatory processes associated with uterine involution, retained placenta, and metritis can elevate core body temperature independently of environmental heat load, thereby generating false-positive alerts in bolus-based heat stress detection systems [32]. Similarly, concurrent infectious diseases—including respiratory infections, coliform mastitis, and postpartum metritis—produce febrile RT elevations that overlap with the RT profile of heat stress and are especially difficult to distinguish during summer months when both phenomena co-occur. Individual-level, event-referenced baseline calibration that accounts for the periparturient transition window is therefore essential for reliable heat stress detection in cows within the first four weeks post-calving. Importantly, continuous RT monitoring during the periparturient period also carries prognostic value: Kovács et al. [43] demonstrated that both rumination time and RT in the days preceding parturition are significant predictors of dystocia in dairy cows, with dystocic cows exhibiting lower rumination activity and elevated RT in the 24–48 h before calving, underscoring the added diagnostic utility of retaining bolus sensors throughout the transition period. More broadly, subclinical metabolic conditions—including subclinical ketosis, hypocalcemia, and displaced abomasum—have been associated with altered RT profiles in the periparturient period, independent of infectious fever or environmental heat load, further underscoring the need for metabolic status covariates in heat stress detection algorithms applied to transition cows [25].

The overarching implication is unambiguous: a single RT threshold applied uniformly across a herd will generate unacceptable false-positive and false-negative rates due to the systematic influences of breed, parity, milk yield, and diurnal rhythm—with a fixed threshold such as 39.5 °C generating false-positive alerts for Jersey cows at near-normal temperatures while simultaneously failing to alert for heat-stressed Holsteins whose individual baseline exceeds the population mean. Individual-level baseline calibration is therefore not a refinement but a prerequisite for reliable heat stress detection—a step tractable with the longitudinal data available from permanently retained boluses but impossible with point-in-time or wearable sensor measurements [40,43]. Stone et al. [7] quantified this variability directly, showing that Holstein cows with higher daily milk yield exhibit a steeper RT response per unit THI increment compared with lower-producing animals, and that multiparous cows consistently showed higher baseline RT than primiparous cows at equivalent milk yield levels. Breed differences were similarly substantial: Jersey cows maintained lower RT than Holsteins across all THI levels, with differences of up to 0.5 °C at peak heat stress—an effect large enough to substantially alter alert rates if a breed-agnostic threshold is applied.

The diagnostic challenge of distinguishing heat stress from fever and estrus using RT data alone underscores the clinical value of multiparametric bolus configurations. While all three conditions elevate RT [7,43,44], their multiparametric signatures are distinct and diagnostically informative. Heat stress produces a gradual RT elevation temporally coupled with rising THI, accompanied by reduced lying time, reduced rumination duration, increased drinking frequency, and—in next-generation devices—elevated heart rate reflecting cardiovascular heat strain [38]. Crucially, the RT elevation under heat stress develops gradually over hours as environmental THI rises—typically 0.3–0.8 °C above an individual thermoneutral baseline within 2–4 h of sustained THI > 68 [7,38]. In contrast, fever associated with infectious disease typically produces a faster (<1 h) and often larger absolute RT rise (>1.0 °C) that is temporally decoupled from ambient THI. Estrus-associated RT elevations are intermediate in magnitude (0.2–0.5 °C) and temporally aligned with the behavioral estrus window [45]. This asymmetric temporal-magnitude fingerprint means that a sliding-window analysis of RT kinetics—rate of rise, peak amplitude, and duration—combined with THI co-monitoring, can disambiguate the three conditions with substantially higher specificity than a single-threshold approach [16,20]. Fever arising from infectious disease produces a faster and often higher absolute RT rise that is temporally decoupled from environmental THI, with variable changes in activity and rumination depending on the pathogen and disease stage [43,45]; Kim et al. [46] demonstrated that mastitis-associated febrile RT profiles were distinguishable from heat stress profiles by their steeper onset slope and sustained elevation independent of ambient conditions. Estrus produces a modest RT elevation (0.3–0.8 °C) coinciding with a characteristic surge in neck-collar activity and, in multi-sensor systems, an increase in standing time—the inverse of the lying time reduction seen in heat stress [45]. Individual baseline calibration, combined with concurrent monitoring of at least activity and drinking behavior, is therefore the minimum analytical requirement for clinically reliable differentiation between these three physiologically distinct causes of RT elevation [17,47].

### 4.2. Long-Term RT Dynamics and Seasonal Patterns

Liang et al. [19] extended this characterization to a substantially larger dataset, monitoring 93 cows across 615 cow-days, encompassing Holstein, Jersey, and crossbred animals. Their analysis confirmed that ambient temperature was a highly significant predictor of RT, with summer values significantly higher than those in spring, autumn, or winter. The mean daily RT across all breeds and seasons was 40.14 ± 0.32 °C, with the same diurnal extremes—nadir at 10:00, acrophase at 23:30—as those documented by Stone et al. [7], attributable to feeding patterns and fermentation dynamics. Crossbred cows again showed significantly lower RT than Holsteins, while the RT response to rising external temperature was more pronounced in higher-producing cows.

Together, Stone et al. [7] and Liang et al. [19] provide an empirical basis for the breed-adjusted, time-normalized interpretation of RT data that underpins the machine-learning frameworks reviewed in subsequent sections. The consistency of findings across studies of different scale—from 36 to 93 cows, and from 10 weeks to 615 cow-days—strengthens confidence in the biological coherence of the RT signal as a heat stress indicator.

## 5. Intraruminal Bolus Technology: From Single-Parameter to Multiparametric Sensing

### 5.1. Evolution of Commercial Bolus Systems

The first-generation commercial intraruminal boluses provided only temperature monitoring, transmitting data via short-range radio protocols to barn-mounted receivers at 10–60 min intervals [48]. Validation studies confirmed RT as a reliable indicator for fever detection, with Kim et al. [46] demonstrating in a 50-cow, six-month field study that subclinical mastitis could be identified 24–48 h before clinical signs appeared, with 14 of 15 subclinical episodes generating automated alerts in advance of diagnosis. Timsit et al. [44] demonstrated the efficacy of RT boluses for early detection of bovine respiratory disease in young bulls, and Rose-Dye et al. [49] documented characteristic RT profiles in experimental BVD and Mannheimia haemolytica infections. These applications established the bolus as a multi-purpose diagnostic instrument.

Second-generation commercial systems integrated tri-axial accelerometry with temperature, enabling simultaneous detection of estrus through activity surges, parturition prediction through changes in lying behavior, and lameness monitoring through altered movement patterns [45,50]. The convergence of multiple high-value detection capabilities in a single, permanently retained device substantially improved the economic case for bolus adoption relative to wearable alternatives (Table 2).

### 5.2. Machine-Learning Integration: The Four-Level Processing Architecture

Convergent advances in sensor hardware, communication protocols, and analytical software have driven the progressive integration of AI methods into bolus-based heat stress monitoring systems. Ammer et al. [20] were among the first to rigorously evaluate reticuloruminal temperature as a heat stress indicator in commercial dairy conditions, studying 28 Holstein–Friesian cows on three farms in Lower Saxony across ten months. They reported a significant positive correlation (r = 0.22) between daily median RT and THI when the THI was above 70, whereas the coefficient ranged between r = −0.08 and +0.06 when THI < 70. They also demonstrated the confounding effects of milk yield and water intake on the RT signal—findings that underscored the need for multivariate contextualization of bolus data and prefigured the individual-calibration frameworks that followed [20]. Levit et al. [47] demonstrated a pivotal application of bolus-derived RT for heat stress management. In a controlled trial with 30 lactating cows in Israel, they proposed a dynamic sensor-based cooling regime in which evaporative cooling was activated and adjusted based on individual RT changes recorded by SmaXtec boluses, rather than by ambient THI schedules [47]. Compared with a conventional time-based control group, sensor-based cows experienced shorter daily heat stress duration (5.03 vs. 9.46 h/day), higher milk fat (3.65 vs. 3.43%), higher milk protein (3.23 vs. 3.13%), and greater energy-corrected milk yield, demonstrating that bolus-guided individual cooling is both technically feasible and productively beneficial. Woodward et al. [14] further extended the scope of bolus-based heat stress analytics to pasture-based systems, using SmaXtec bolus data from 443 cows across three New Zealand farms. They defined a herd-level heat stress rate (HSR) metric as the proportion of cows with scaled RT more than 3 SD above their individual mean—explicitly acknowledging that a fixed RT threshold is insufficient given between-animal variation—and developed machine-learning models (linear model, generalized additive model, cubist) to predict HSR from automated weather station data, achieving promising predictive accuracy (HSR > 25% threshold) and demonstrating the feasibility of weather-forecast-driven proactive heat stress alerts in outdoor dairy systems. Hajnal et al. [17] conducted a systematic review of AI methodologies applicable to bolus-derived data. They proposed a four-level hierarchical processing architecture that integrates and formalizes the analytical approaches implicit in the above studies. The first level encompasses on-device signal conditioning—noise filtering, baseline correction, and data compression—constrained by the energy budget imposed by the primary battery. The second level covers gateway-level aggregation, timestamping, and packet integrity verification. The third level involves the farm management software, which executes real-time event detection and alerting using rule-based or lightweight machine-learning models calibrated to individual animals. The fourth level—cloud-based analytics—applies computationally intensive models to longitudinal datasets for trend analysis, herd-level prediction, and model updating.

This architecture reflects a principled engineering response to the tension between computational depth and energy constraint: the most sophisticated models run offline on accumulated data. In contrast, the time-critical alerting functions run as lightweight classifiers on the edge. For heat stress monitoring specifically, Hajnal et al. [17] identify the Random Forest algorithm as particularly well-suited to third-level real-time alerting, citing its tolerance for missing data arising from communication packet loss—a practically important property given that packet loss rates of 5–15% are typical in commercial barn deployments. Long short-term memory (LSTM) networks are well-suited for fourth-level temporal modeling of heat stress dynamics, where the ability to capture multi-hour lagged dependencies between THI exposure and RT elevation is analytically valuable. The authors also emphasize that machine-learning models trained on raw, uncorrected RT data show markedly degraded performance relative to models trained on drinking-water-corrected signals, making effect removal a prerequisite rather than an optional refinement [17].

The architectural separation of real-time alerting from longitudinal trend analysis has important implications for heat-stress-specific pipeline design. Real-time alerting at level three must operate within the latency constraints of cooling interventions—typically 30–60 min for evaporative cooling to meaningfully reduce core temperature —limiting on-farm inference complexity. Level-four cloud analytics, by contrast, can apply computationally intensive approaches, including LSTM-based sequence modeling, transfer learning for cross-farm calibration, and genomic covariate integration, without real-time constraints. The most practically demanding component—early individual alerting before THI-triggered barn systems respond—is thus handled by a lightweight Random Forest classifier at level three. At the same time, the more scientifically complex tasks of understanding seasonal acclimatization trajectories and predicting future heat stress risk from forecast weather data are delegated to level four.

**Figure 5:**
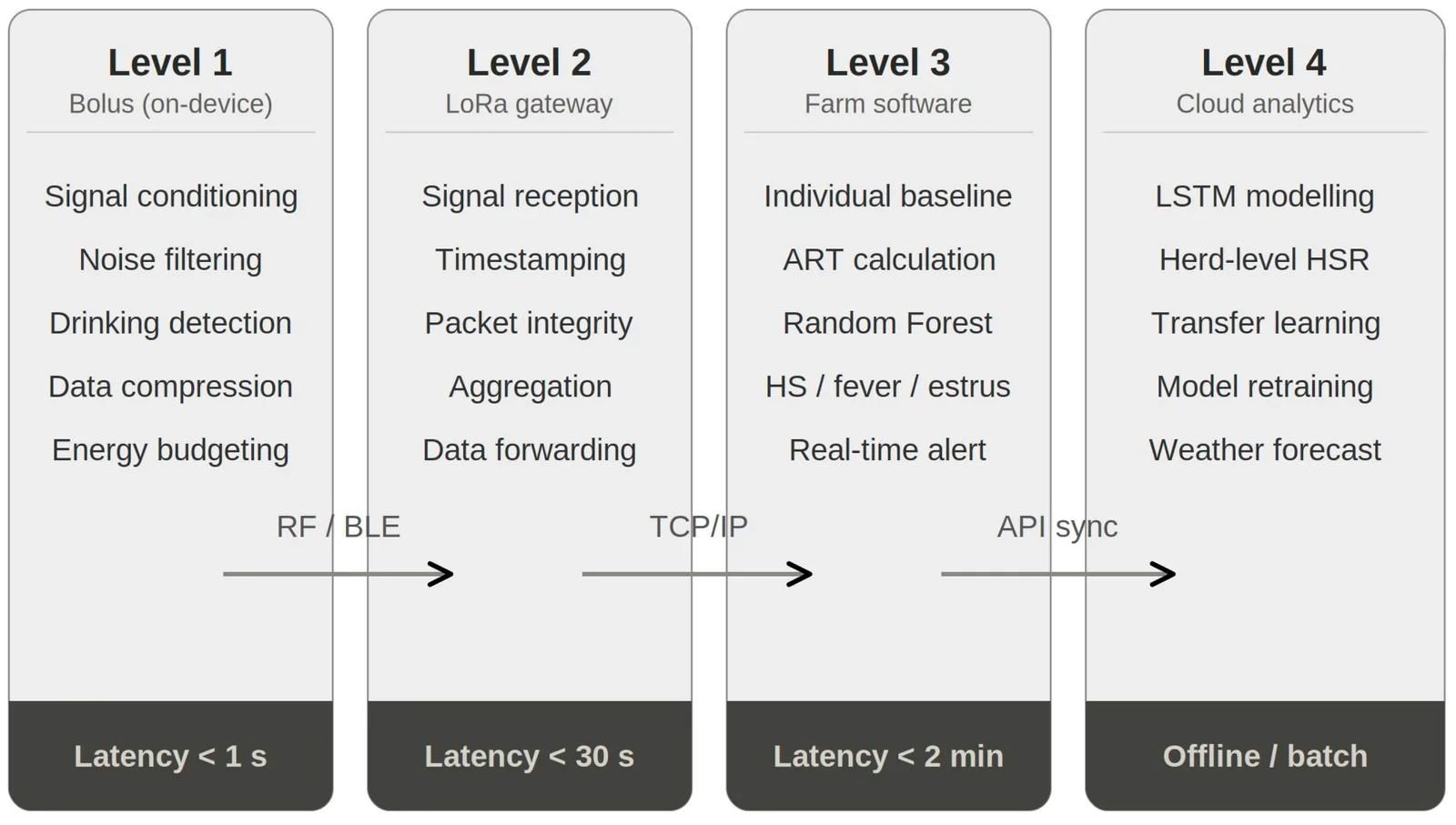
Four-level machine-learning processing architecture for intraruminal bolus-derived data proposed by Hajnal et al. [17]. ART = adjusted reticuloruminal temperature; BLE = Bluetooth Low Energy; HSR = herd-level heat stress rate; IBI = inter-beat interval; LSTM = long short-term memory; RF = radio frequency.

Burnett and Cerri [45], studying 190 estrous episodes, demonstrated that individual baseline-referenced metrics, specifically, the area under the RT curve relative to a rolling seven-day non-event baseline, substantially outperformed fixed-threshold approaches for thermal event detection, consistent with the individual-calibration principle advocated by Hajnal et al. [17] and the empirical findings of Stone et al. [7]. The complete four-level scheme—spanning on-device signal conditioning, gateway-level aggregation, farm-level real-time alerting, and cloud-based analytics—is summarized in Figure 5.

### 5.3. Multiparametric Bolus Development

**Figure 6:**
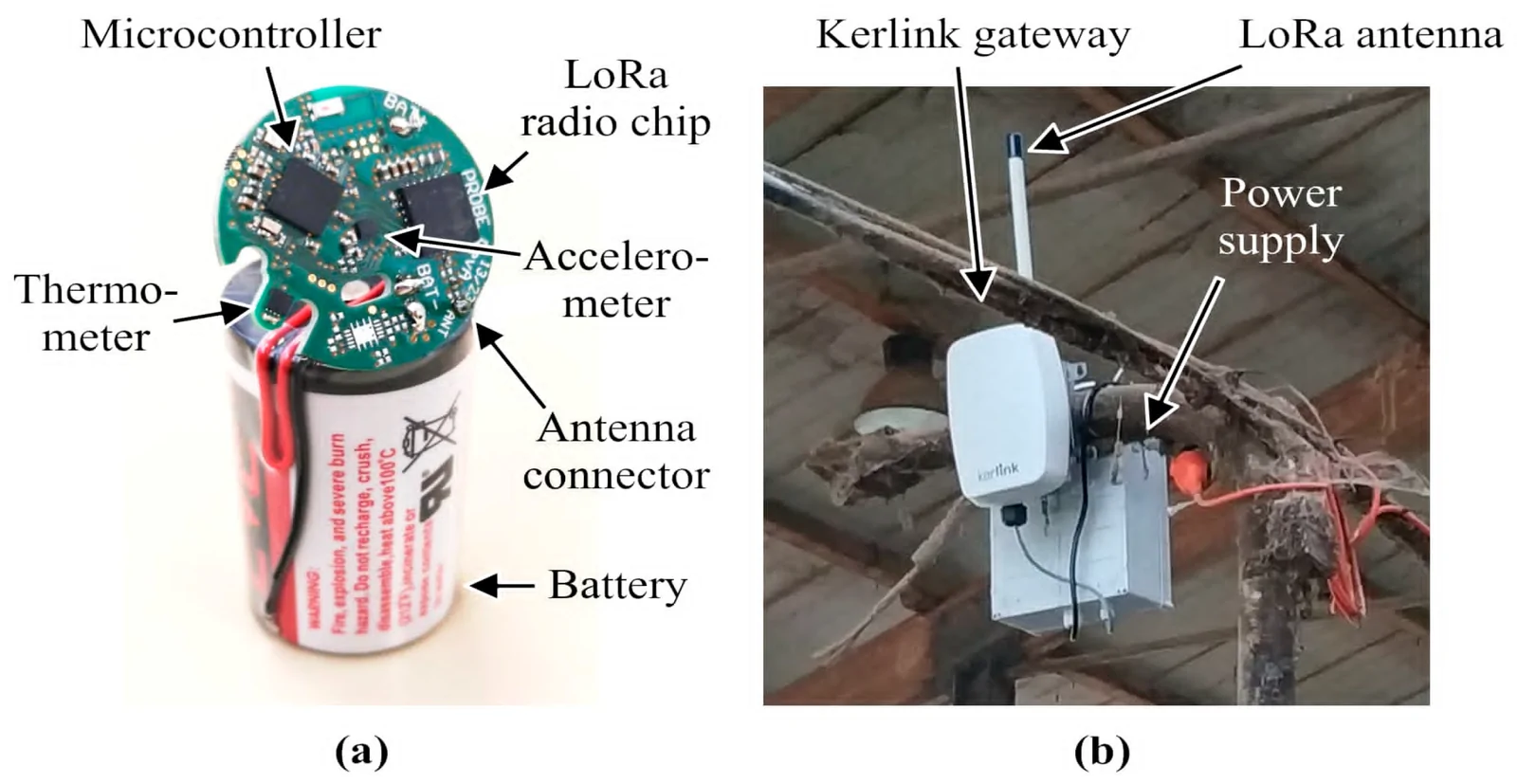
The hardware components (printed circuit board of the bolus with the high capacity battery (**a**) and the gateway installed under the roof of the barn (**b**)) of the versatile lifetime intraruminal bolus sensor developed by Vakulya et al. [16].

The most technically advanced published development of the multiparametric intraruminal bolus concept was reported by Vakulya et al. [16], who described the full design and a field validation process for a versatile lifetime bolus sensor combining temperature measurement, tri-axial microelectromechanical systems (MEMS) accelerometry, pulse-rate monitoring, drinking behavior detection, and LoRaWAN data transmission in a single device. The mechanical design—40 mm diameter, 120 mm length, polyoxymethylene copolymer housing—satisfies both the anatomical requirements for reticulum retention and the material requirements for resistance to the acidic, enzymatic, and mechanically abrasive ruminal environment. The non-rechargeable D-size lithium-thionyl-chloride primary battery provides an estimated operational lifetime of approximately 14 years at LoRaWAN SF12 spreading factor settings, substantially exceeding the 4–6-year lifetime of current commercial systems. The above structure contrasts with commercial platforms such as SmaXtec Classic—which integrates temperature, activity, rumination, and drinking monitoring via Bluetooth transmission and cloud-based AI analytics [14,26]—and has been the most widely used bolus platform in published heat stress research to date [20,39,47]. The SmaXtec system’s cloud-based TruD^®^ platform applies proprietary AI algorithms to individual cow data, and its 10 min measurement interval, combined with built-in drinking-event detection, makes it well-suited to the individual RT-based heat stress analyses demonstrated by Levit et al. [47] and Woodward et al. [14]. The Bella Ag calf bolus (Bella Ag LLC, Loveland, CO, USA) represents a parallel development trajectory, with ±0.25 °C accuracy and designed for pre-weaning calves, further illustrating the diversification of intraruminal sensor platforms across bovine life stages [26]. Recent studies have also demonstrated the utility of multi-sensor boluses that include pH sensing alongside temperature and accelerometry, enabling concurrent monitoring of subacute ruminal acidosis risk—a condition that is both elevated by heat-stress-induced reductions in dry matter intake and potentially confounded with the RT signal through altered fermentation dynamics [18,26]. The breadth of this emerging hardware ecosystem (Figure 6) underscores the importance of the standardized validation frameworks called for by Vakulya et al. [16] and Hajnal et al. [17]; without harmonized protocols, the performance of different bolus platforms on heat stress detection cannot be systematically compared across studies.

The incorporation of pulse-rate sensing is a notable advance that has not previously been documented in intraruminal devices. Cardiac output and heart-rate increase during heat stress to meet the dual demands of enhanced peripheral blood flow for cutaneous heat dissipation and sustained mammary gland perfusion [51]. Heart rate thus offers a direct measure of cardiovascular heat strain, complementing the thermal indicator provided by RT and the behavioral indicators provided by accelerometry. The concurrent availability of RT, activity, rumination duration, drinking frequency, and pulse rate in a single device that is never lost, misplaced, or removed opens the possibility of comprehensive physiological profiling across the full range of heat stress severity. Field testing of the Vakulya et al. [16] device needs published validation against video-based behavioral observation and Polar (or ECG) heart-rate sensors as references. The LoRaWAN protocol was selected over NB-IoT because farm-deployed gateways can be positioned to optimize signal coverage using RSSI mapping and remain under full operator control, without relying on cellular network coverage that may be unreliable in rural settings. Earlier field observations by the same research group [52] provided initial evidence for the practical utility of long-term rumen temperature analysis in commercial dairy settings, forming part of the empirical foundation for the more comprehensive sensor development.

## 6. Heat Stress Detection Algorithms for Bolus-Derived Data

### 6.1. Threshold-Based and Baseline-Adjusted Approaches

The simplest heat stress detection algorithm uses a fixed population-level RT threshold—typically 39.5 °C or 40.0 °C—and generates an alert whenever it is exceeded. While operationally simple, this approach is undermined by the documented inter-animal variation in baseline RT [7,19] and the systematic influences of breed, parity, time of day, and season (Table 3). A threshold calibrated to the mean of a heterogeneous herd will be simultaneously too sensitive for high-baseline individuals and insufficiently sensitive for low-baseline individuals. Ammer et al. [20] quantified this problem empirically; in their multi-farm study, the THI threshold at which RT began to increase was 65, and the magnitude of the RT–THI relationship varied substantially with milk yield level, confirming that no single fixed RT threshold is appropriate across a herd with diverse production characteristics. Levit et al. [47] operationalized this insight in their sensor-based cooling trial, in which the RT threshold triggering cooling activation was adjusted weekly for each cow based on the previous week’s individual RT trajectory, rather than being set as a fixed value. This approach directly reduced heat stress duration compared with the conventional THI-scheduled control group.

Individual baseline calibration addresses this limitation by computing a rolling reference temperature for each cow—typically the mean of the preceding 5–10 days under thermoneutral conditions, excluding drinking-water effect periods—and expressing the current RT as a deviation from this individual reference [14,45]. Burnett and Cerri [45] demonstrated the superiority of this approach for estrus detection, and the principal transfer directly to heat stress. Lees et al. [39] operationalized this concept for heat stress prediction in grazing cows, showing that ART-based individual deviation metrics substantially outperformed raw RT thresholds for event detection, and that a machine-learning classifier trained on ART features achieved further improvement over threshold-based ART approaches alone. Džermeikaitė et al. [53] further demonstrated that machine-learning models trained on reticuloruminal pH and temperature data from smaXtec boluses achieved superior discrimination of heat stress events compared with fixed-threshold approaches, while simultaneously characterizing effects on milk quality and rumination behavior, confirming the value of multivariate bolus-derived feature sets. Woodward et al. [14] extended the individual-calibration concept to the herd level in pasture-based systems, defining their HSR metric as the proportion of cows with scaled RT more than 3 SD above each cow’s own mean. This formulation inherently accounts for individual baseline differences while producing a single, interpretable herd-level indicator. Their finding that a fixed threshold of 39.5 °C was insufficient to indicate heat stress uniformly across all animals further confirms the inadequacy of population-level fixed thresholds. The HSR approach also facilitates the integration of bolus data with forecast weather variables (air temperature, solar radiation, humidity, wind speed) to develop predictive alert models up to several hours in advance of expected heat stress events—a practically important capability for pasture-based systems where real-time environmental management is more limited than in housed herds.

### 6.2. Machine-Learning Classifiers for Heat Stress Events

Supervised machine-learning classifiers trained on labeled bolus data offer greater discriminative power than threshold methods by exploiting non-linear feature interactions and temporal structure. The feature space for heat stress classification includes current and lagged RT values; rolling RT mean, variance, and rate of change; accelerometer-derived lying time, bout count, and rumination duration; drinking episode frequency and duration; time of day; ambient THI; and individual covariates such as breed, parity, and days in milk [14,17,54]. Woodward et al. [14] compared linear, generalized additive, and cubist models (a piecewise linear regression tree) for predicting HSR from weather variables, finding that all three achieved usable predictive accuracy. However, they recommended further testing across a wider environmental range before operational deployment. The cubist model—a piecewise linear regression with decision-tree splits—showed particular promise for capturing the non-linear relationship between weather variables and heat stress incidence in New Zealand pasture systems. Levit et al. [47] implicitly applied a simpler adaptive learning approach: their sensor-based cooling regime adjusted RT thresholds weekly based on each cow’s measured thermal response from the preceding week, constituting a rolling, cow-specific recalibration that outperformed the static THI-scheduled control. While not a formal machine-learning classifier, this adaptive individualization embodies the same core principle as more sophisticated algorithmic approaches: the optimal threshold for heat stress detection is unique to each animal and shifts over time. This approach was validated by Džermeikaitė et al. [53], who applied ML models to reticuloruminal pH response data from commercial boluses, demonstrating that multiparametric bolus-derived features including pH outperform univariate RT metrics in classifying heat stress severity.

Random Forest classifiers are well-suited to this heterogeneous, partially correlated feature set and handle missing data gracefully—a critical property for real-time bolus data where packet loss is routine [17]. For temporal modeling, LSTM networks capture the multi-hour dynamics of heat stress onset and recovery, which unfold on timescales longer than the inter-measurement interval of most bolus systems. When pulse-rate data from next-generation devices, such as those described by Vakulya et al. [16], are included in the feature set, the discriminative information available to the classifier is substantially enriched, since heart rate provides direct evidence of cardiovascular strain that RT alone cannot capture. A persistent analytical challenge is the class imbalance inherent in heat stress datasets, as severe heat stress events are relatively rare compared with thermoneutral periods, biasing classifier training towards the majority class. Techniques including synthetic minority oversampling, cost-sensitive learning, and anomaly-detection framing have been applied with encouraging results, though direct validation in large dairy field datasets remains limited [17]. In practice, these approaches differ in what they require from the data. Synthetic minority oversampling generates artificial heat stress observations in feature space, but for bolus time series the synthetic samples must be created within animal and within time block, otherwise interpolation across cows or across adjacent windows introduces leakage between the training and test sets and inflates apparent performance. Cost-sensitive learning avoids this by leaving the data unchanged and instead weighting the minority class inversely to its prevalence—implemented as class weights in Random Forest or as a weighted loss in LSTM training—which is the more conservative option when the number of confirmed severe heat stress events per herd is small. Anomaly-detection framing sidesteps the labeling problem altogether by modeling each cow’s thermoneutral RT profile and flagging departures from it, which fits the individual-calibration logic described above but provides no severity grading. Whichever strategy is chosen, model performance should be reported as precision–recall characteristics or sensitivity at a fixed false-alarm rate rather than as overall accuracy, since a classifier that never predicts a heat stress event can still achieve high accuracy in an imbalanced dataset [17].

### 6.3. Integration of Bolus Data with Environmental and Production Records

Bolus RT data realize their full interpretive value when analyzed alongside environmental THI measurements, farm management records, and individual production data. Stone et al. [7] demonstrated that the RT response to a given THI value is substantially moderated by breed, milk yield, and parity—a Holstein at peak lactation shows a larger RT increase per unit THI than a Jersey in mid-lactation—and algorithms failing to account for these covariates will generate systematically biased alerts. Ammer et al. [20] similarly showed that the THI–RT relationship varied with milk yield across a multi-farm German cohort, and that drinking-water intake frequency was a significant confounding variable that required explicit modeling. Seamless data integration among the bolus platform, the THI sensor network, and herd management software is technically feasible but organizationally demanding, particularly on smaller farms with heterogeneous data infrastructure [17,55]. Woodward et al. [14] provided a proof-of-concept for this integration in pasture systems, combining SmaXtec RT data with automated weather station records across three farms and over two years, and specifically highlighted that heat stress indices validated for cattle—including THI—have limitations for cows exposed to wind and solar radiation, underscoring the importance of system-specific model calibration.

Interpreting RT data in its full individual context is not a statistical refinement, but a physiological necessity. The RT response to a given THI increment is moderated by the cow’s current energy balance status, which determines the proportion of metabolic heat that is fermentation-derived versus mammary-metabolic in origin [28]. A cow in severe negative energy balance in early lactation may show a smaller RT response to rising THI than a mid-lactation cow at the same production level, because reduced dry matter intake constrains fermentation heat production even as the animal is simultaneously heat-stressed. Integrating days-in-milk and body condition score as covariates—alongside the breed, parity, and milk yield covariates identified by Stone et al. [7]—would address this confounding pathway. Nardone et al. [56] further highlight that heat stress impacts on livestock will intensify under climate change projections, increasing the economic urgency of robust, multi-covariate individual monitoring frameworks validated across the thermal environments and production systems that will characterize future dairy agriculture.

## 7. Bolus-Based Heat Stress Monitoring

### 7.1. Heat Stress Detection and Cooling Intervention Timing

The primary application of bolus-based heat stress monitoring is the real-time detection of thermal stress events and triggering targeted cooling interventions. Conventional barn cooling systems—sprinklers, fans, and shade—are typically activated by barn-level THI thresholds rather than individual animal temperature. The availability of individual RT enables a more targeted approach: cows whose RT exceeds personalized alert thresholds can be prioritized for cooling resources, and intervention can be timed relative to the physiological onset of heat strain rather than the environmental conditions that precede it [29]. Collier et al. [29] demonstrated that individual-level temperature monitoring can identify heat-stressed animals up to three hours earlier than THI-based barn monitoring, a temporal advantage that is practically meaningful for interventions such as pre-cooling before peak afternoon heat. This early detection benefit extends beyond the lactating herd: Tao et al. [32] showed that cooling dry cows during late gestation generated sustained benefits in subsequent lactation milk yield, immune function, and calf health—a population entirely invisible to production-based monitoring systems, yet among the most physiologically vulnerable to heat stress. Bolus-based RT monitoring thus broadens the reach of heat stress management to this critical group and, by identifying heat-stressed animals up to 3 h earlier than THI-based barn monitoring [29], has the potential to meaningfully reduce the cumulative duration of above-threshold RT exposure and thereby limit carryover production losses. Beyond individual intervention, continuous RT monitoring also enables identification of heat-stress-sensitive cows at the herd level, allowing targeted grouping and tailored management of the most thermally susceptible animals. This stratified approach has direct implications for animal breeding and genetic selection for thermotolerance. It contributes to the sustainability of the farming operation by reducing unnecessary energy consumption in cooling systems and water use in evaporative cooling strategies.

The transition period is associated with physiological immunosuppression, negative energy balance, and heightened susceptibility to both infectious and metabolic disease [33]. Heat stress during this period compounds these risks: Tao et al. [32] showed that prenatal heat stress impairs neonatal immune function and reduces subsequent lactation yield even after the dam has recovered, demonstrating intergenerational effects not captured by production records alone. Bolus sensors retained through the dry period and periparturient transition provide continuous individual RT monitoring during this critical window, enabling differentiation of the normal postpartum RT elevation—which may reach 39.5–40.0 °C in the first 24–48 h after calving due to uterine involution—from a heat stress-associated elevation that warrants cooling intervention.

### 7.2. Reproductive Management

The integration of heat stress detection with reproductive management offers economically significant benefits. Because estrus and heat stress both elevate RT, their discrimination requires multiparametric context: estrus is typically associated with a simultaneous rise in activity and a modest RT elevation of 0.3–0.8 °C, without the lying-time reduction, increased drinking, or respiratory rate changes that characterize heat stress [7,45]. Bolus systems that monitor RT, activity, and drinking behavior concurrently can apply multi-feature classifiers to discriminate among these states, enabling both estrus detection and heat stress alerting within a single analytical framework. The economic value of preventing insemination during heat stress periods—when conception rates may be halved—adds substantially to the return on investment from bolus monitoring [30,31].

The practical implementation of this discrimination requires a classifier that is robust to real-world variability in bolus data quality, including packet loss, drinking-water effects, and individual RT baseline drift. In the Levit et al. [47] trial, sensor-based individual cooling did not impair estrus detection rates relative to the control group, confirming that the two monitoring objectives can be pursued concurrently. The combination of RT-based heat stress detection and activity-based estrus identification within a single bolus system, therefore, represents one of the most economically compelling value propositions in precision dairy health management.

### 7.3. Disease Surveillance in the Context of Thermal Stress

Heat stress is immunosuppressive, increasing susceptibility to infectious diseases such as mastitis, respiratory infections, and postpartum metritis. The bolus provides simultaneous monitoring of the thermal environment and its physiological consequences, enabling differentiation between fever and heat stress—both of which elevate RT, but through different mechanisms and with distinct behavioral and cardiovascular profiles. Kim et al. [46] demonstrated that mastitis-associated fever produced faster and higher RT elevations than heat stress alone, and that automated RT-based alerting identified 14 of 15 subclinical mastitis episodes before clinical signs. In the context of heat stress, where the baseline RT is already elevated, distinguishing an additional febrile component requires individual calibration and a multiparametric context that modern bolus systems are designed to provide [17].

The immunosuppressive effect of heat stress operates through multiple pathways relevant to the interpretation of bolus RT data. Elevated core temperature directly impairs neutrophil and lymphocyte function, reduces immunoglobulin synthesis, and promotes a pro-inflammatory cytokine environment, increasing susceptibility to Gram-negative bacterial infections, including coliform mastitis and postpartum metritis [33]. This immunosuppression manifests in the bolus RT signal as a reduced febrile response to infection: a heat-stressed cow with an early mastitis infection may show a smaller absolute RT elevation than a thermoneutral cow with the same pathogen load, because her baseline RT is already elevated and her thermoregulatory reserve is depleted. This compression of the diagnostic RT window under heat stress conditions means that alert thresholds for disease detection must be calibrated to the individual’s current thermal status, not an absolute population-level value. The individual-calibration framework advocated for heat stress detection [7,17] is therefore equally essential for disease surveillance in hot weather, and the two applications are analytically inseparable.

## 8. Methodological Challenges and Standardization

Despite substantial progress, several methodological challenges limit the translation of bolus-based heat stress monitoring from experimental validation to reliable farm-scale deployment. The drinking-water effect, although algorithmically addressable [38,39], requires robust detection methods that function reliably across the variable drinking patterns of individual cows in different housing systems. Fermentation-induced RT fluctuations require analogous treatment for high-precision applications.

A sensor accuracy of ±0.1–0.3 °C is adequate for fever detection but limits sensitivity to smaller thermal signals associated with early mild heat stress and estrus. Vakulya et al. [16] address this through an expanded calibration protocol, and ongoing advances in MEMS thermometry are progressively reducing measurement uncertainty. Communication reliability—with typical packet loss rates of 5–15% in commercial barn deployments [17]—remains a practical limitation for time-series models sensitive to data gaps; robust imputation and gap-tolerant classifiers are essential features of production-grade systems.

Perhaps most critically, the absence of standardized, crossbreed, cross-climate validation protocols for bolus-based heat stress monitoring systems makes evidence-based comparison between commercial and research systems difficult. Vakulya et al. [16] identify this as a priority gap, and the development of PLF validation standards—including heat-stress-specific protocols—is urgently needed. The performance of individual components (sensor accuracy, communication reliability, algorithm sensitivity) and of integrated systems must be characterized separately and together across the full range of commercial conditions, including different breeds, management systems, and climate zones. Given the diversity of instruments, the need for standardization is quite obvious. Proposing specific steps for standardization is beyond this review. Therefore, we refer to existing initiatives, such as the validation initiatives and protocols of the International Committee for Animal Recording (ICAR). The ICAR validates PLF technologies to ensure sensor data and automated systems meet rigorous international performance standards [57]. Sensors and other technologies should be tested in more diverse farming environments (i.e., not only in experimental farm conditions), across different management systems (e.g., intensive dairy production, pasture-based systems, freestall and tiestall housing) and geographical locations with diverse climatic conditions, in different breeds, and in animals of different ages and in varied physiological stages (e.g., lactation stage, reproductive stage) [58]. Translating these requirements into commercially ready systems depends on three parallel developments. First, the analytical layer must be engineered as a maintained pipeline rather than a fixed model. Gap-tolerant preprocessing and imputation, versioned models with documented training cohorts, periodic recalibration against newly labeled events, and monitoring for performance drift as herd composition, ration, and climate change over time—the four-level edge-to-cloud architecture described in Section 5 provides a practical template for allocating these functions between device, gateway, and server. Second, field data integration requires that bolus outputs be delivered into the systems farms already use—herd management software, milk recording databases, and cooling controllers—through documented interfaces and shared data formats, so that RT-derived alerts appear alongside production and health records rather than in a separate proprietary portal [55]; the sensor-to-actuator pathway demonstrated by Levit et al. [47] illustrates what becomes possible once this integration is achieved. Third, farmer acceptance depends less on classifier accuracy than on the operational cost of false alarms and on whether each alert maps to a defined action: alert volume must be tuned to the number of interventions a farm can realistically absorb, thresholds should be adjustable at farm level, and outputs should be expressed as interpretable quantities—duration of heat load per cow, proportion of the herd affected—rather than as model scores. Independent performance verification under recognized protocols [57,58] supports all three, since farms and advisers require comparable evidence of accuracy before committing to a multi-year investment in bolus infrastructure.

## 9. Future Research Directions

An overarching context for all five research priorities is the projected intensification of heat stress under climate change. Mean annual THI values across major dairy-producing regions of Europe, North America, and Australasia are projected to increase by 3–8 units by 2100, with severe heat stress events (THI > 80) increasing 3- to 5-fold in currently temperate zones [2,3,59]. Breed-specific RT alert thresholds validated under current conditions will require recalibration as baseline ambient temperatures rise, and within-breed variation in thermotolerance—currently masked by modest heat loads in temperate climates—will become increasingly relevant [11,42]. Longitudinal bolus RT datasets spanning multiple years offer a practical resource for tracking these secular changes and enabling prospective threshold recalibration—a capability not available with point-in-time or wearable sensor measurements [16,17].

Six research priorities emerge from this review. First, large-scale, multi-breed field validation of drinking-water-corrected, individual baseline-adjusted RT as a heat stress indicator—using rectal temperature loggers, respiratory rate scoring, and THI records as reference measures—is needed to establish sensitivity and specificity under commercial conditions. The field validation program associated with Vakulya et al. [16] represents a step in this direction.

Second, validation of bolus-based pulse-rate measurement in adult lactating cows is a high-priority task. Heart rate or heart-rate variability provides cardiovascular evidence of heat strain [42,51,60,61], and its accuracy relative to electrocardiographic reference systems under both thermoneutral and heat stress conditions must be established before operational deployment.

Third, applying transfer learning and domain adaptation techniques to bolus-based heat stress models would reduce the farm-specific calibration data requirements that currently limit scalability. A model pre-trained on well-characterized breed cohorts could be fine-tuned to a new farm with limited historical data—reducing the calibration period from months to days—since the RT–THI relationship is physiologically consistent across farms of the same breed composition. However, it differs in slope and intercept due to local acclimatization and management practices.

Fourth, within-breed genetic variation in thermotolerance has not been systematically characterized using bolus RT as the phenotypic endpoint, despite documented breed differences in the RT response to THI [7,11,19]. Establishing this relationship would provide a high-throughput phenotyping tool for thermotolerance selection, with advantages over rectal temperature or respiratory rate measurements in terms of data continuity and operator independence.

Fifth, embedding bolus data streams within farm decision-support platforms requires investment in data interoperability standards, API integration between bolus platforms and herd management software, and interpretable model outputs accessible to farm personnel without specialist expertise [17,55]. For heat stress monitoring, this includes coupling RT alerts with automated cooling system controllers—enabling sensor-to-actuator pathways of the type demonstrated by Levit et al. [47]—and standardizing communication protocols between bolus platforms and building management systems, an area where industry standards are currently lacking.

Sixth, although the focus of this review is heat stress, the year-round retention of boluses means the same sensor infrastructure can capture RT responses under cold-stress conditions, which are becoming increasingly relevant as climate change generates more frequent and severe temperature extremes in both summer and winter. Systematic collection of RT data under cold conditions would expand the utility of bolus-based monitoring beyond heat stress to a broader thermal welfare and production management framework.

## 10. Conclusions

Several alternative biosensor approaches exist for measuring reticuloruminal temperature. The multi-sensor boluses provide a promising platform for continuous, individual-level heat stress monitoring in cattle, combining stable anatomical placement with core temperature measurement shielded from external interference and expanded multiparametric sensing. Reliable heat stress detection requires individual baseline calibration rather than population thresholds, as breed, parity, milk yield, time of day, and season all significantly influence reticuloruminal temperature. Machine-learning frameworks enable the translation of raw sensor signals into actionable farm management decisions. Key remaining challenges, such as drinking-water effect correction, sensor precision, communication reliability, and the absence of standardized validation protocols, are tractable and the subject of active research. As climate projections consistently point to intensification of heat stress risk for dairy production systems worldwide, the development, validation, and deployment of bolus-based individual heat stress monitoring represents one of the most practically consequential challenges facing precision livestock farming.

## Figures and Tables

**Table 1:** Summary of studies reporting reticuloruminal temperature (RT) and pH findings in dairy cattle under heat stress conditions using intraruminal bolus sensors.

Reference	n	Breed	Condition	Key RT Finding	Key pH Finding
Stone et al., 2017 [7]	88	HF/Jersey /crossbred	Field, summer	RT rose 0.3–0.8 °C above THI 65; circadian amplitude preserved	Not reported
Liang et al., 2013 [19]	18	HF	Climate chamber	RT peaked 22:00–23:00 h under HS; nadir shifted vs. thermoneutral	Not reported
Ammer et al., 2016 [20]	438	HF	Multi-farm, Germany	Median RT correlated with THI above 65; SensoTech bolus	Not reported
AlZahal et al., 2008 [21]	6	HF	Climate chamber	RT elevated ~0.5 °C at THI 68–74	pH < 5.8 prolonged post-feeding under HS; drinking artifact characterized
Antanaitis et al., 2023 [22]	75	HF	Commercial farm, summer	RT elevated at THI ≥ 72; drinking-corrected baseline applied	pH decreased significantly at THI ≥ 72; acidosis risk increased
Antanaitis et al., 2022 [23]	60	HF	Commercial farm	RT positively correlated with methane emission proxy	Lower pH associated with higher HS risk; pH 6.22–6.42 optimal
Cantor et al., 2018 [24]	16	HF/crossbred	Experimental drenching	RT dropped; recovery up to 103 min depending on water volume/temp	Not reported
Szalai et al., 2025 [25]	60	HF	Commercial farm, heat stress + postpartum	RT elevated above THI 72; higher RT in cows with postpartum disease; RT correlated with rumination time	Not primary focus; pH decrease observed at THI ≥ 72 in diseased cows

HF = Holstein–Friesian; HS = heat stress; RT = reticuloruminal temperature; THI = temperature–humidity index.

**Table 2:** Overview of commercial and prototype intraruminal bolus platforms used for heat stress monitoring in dairy cattle.

Platform	Manufacturer	Sensors	Communication	Interval	HS Validated	Reference
smaXtec bolus	smaXtec animal care technology, Graz, Austria	RT, pH, activity	LoRa	10 min	Yes	Antanaitis et al., 2022, 2023 [22,23]
Bella Ag bolus	Bella Ag LLC, Loveland, CO, USA	RT, disease marker (core temperature)	Proprietary RF	Configurable	Partial (disease/fever)	Dyer et al., 2016 [26]
University of Óbuda-Albacomp RI Ltd. bolus	University of Óbuda-Albacomp RI Ltd., Székesfehérvár, Hungary	RT, activity, LoRa radio	LoRaWAN	Configurable	Under validation	Vakulya et al., 2024 [16]
SensoTech bolus	Medria/SensoTech, Elven, France	RT	RFID/radio	≥15 min	Partial	Ammer et al., 2016 [20]
AlZahal pH bolus	University of Guelph, Guelph, ON, Canada	RT, pH	Proprietary RF	10 min	Yes	AlZahal et al., 2008 [21]

RT = reticuloruminal temperature; HS = heat stress; LoRa = Long Range radio; LoRaWAN = LoRa Wide Area Network; RFID = radio-frequency identification; RF = radio frequency. Interval = nominal data transmission interval. HS validated = whether the platform has been explicitly used to detect or characterize heat stress in peer-reviewed publications. In these testing environments, LoRaWAN connectivity enabled reliable data transmission over distances of up to several kilometers with minimal power consumption.

**Table 3:** Challenges of using fixed reticulorumen temperature (RT) or individualized baselines.

Metric/Approach	Fixed RT Thresholds (e.g., >39.5 °C)	Individualized Baselines (Machine Learning)
Sensitivity	Low; misses vulnerable cows with lower baseline limits.	High; detects subtle deviations from normal self-history.
False-alarm rate	High; triggered easily by normal post-feeding fermentation spikes.	Low; filters out normal individual physiological spikes.
Early warning window	Late; only triggers after severe systemic heat load occurs.	Early; catches predictive behavioral and metabolic shifts early.

## Data Availability

No new data were created or analyzed in this study. Data sharing is not applicable to this article.

## Abbreviations

The following abbreviations are used in this manuscript:

AI	Artificial intelligence
ART	Adjusted reticuloruminal temperature
HSR	Herd-level heat stress rate
ICAR	International Committee for Animal Recording
LoRaWAN	Long Range Wide Area Network
LSTM	Long short-term memory
PLF	Precision livestock farming
RF	Radio frequency
RT	Reticuloruminal temperature
SARA	Subacute ruminal acidosis
THI	Temperature–humidity index
